# A systematic review of randomized controlled trials on efficacy and safety of transcranial direct current stimulation in major neurodevelopmental disorders: ADHD, autism, and dyslexia

**DOI:** 10.1002/brb3.2724

**Published:** 2022-08-08

**Authors:** Mohammad Ali Salehinejad, Elham Ghanavati, Benedikt Glinski, Amir‐Homayun Hallajian, Anita Azarkolah

**Affiliations:** ^1^ Department of Psychology and Neurosciences Leibniz Research Centre for Working Environment and Human Factors Dortmund Germany; ^2^ Department of Psychology Ruhr‐University Bochum Bochum Germany; ^3^ Department of Psychology University of Tehran Tehran Iran; ^4^ Shariati Hospital Tehran University of Medical Sciences Tehran Iran; ^5^ Atieh Clinical Neuroscience Center Tehran Iran

**Keywords:** ADHD, autism spectrum disorder, dyslexia, neurodevelopmental disorders, RCT, transcranial direct current stimulation, transcranial electrical stimulation, systematic review

## Abstract

**Objective:**

Among the target groups in child and adolescent psychiatry, transcranial direct current stimulation (tDCS) has been more applied in neurodevelopmental disorders specifically, attention‐deficit hyperactivity disorder (ADHD), autism spectrum disorder (ASD), and dyslexia. This systematic review aims to provide the latest update on published randomized‐controlled trials applying tDCS in these disorders for evaluating its efficacy and safety.

**Methods:**

Based on a pre‐registered protocol (PROSPERO: CRD42022321430) and using the PRISMA approach, a literature search identified 35 randomized controlled trials investigating the effects of tDCS on children and adolescents with ADHD (*n* = 17), ASD (*n* = 11), and dyslexia (*n* = 7).

**Results:**

In ADHD, prefrontal anodal tDCS is reported more effective compared to stimulation of the right inferior frontal gyrus. Similarly in ASD, prefrontal anodal tDCS was found effective for improving behavioral problems. In dyslexia, stimulating temporoparietal regions was the most common and effective protocol. In ASD and dyslexia, all tDCS studies found an improvement in at least one of the outcome variables while 64.7% of studies (11 of 17) in ADHD found a similar effect. About 88% of all tDCS studies with a multi‐session design in 3 disorders (16 of 18) reported a significant improvement in one or all outcome variables after the intervention. Randomized, double‐blind, controlled trials consisted of around 70.5%, 36.3%, and 57.1% of tDCS studies in ADHD, ASD, and dyslexia, respectively. tDCS was found safe with no reported serious side effects in 6587 sessions conducted on 745 children and adolescents across 35 studies.

**Conclusion:**

tDCS was found safe and partially effective. For evaluation of clinical utility, larger randomized controlled trials with a double‐blind design and follow‐up measurements are required. Titration studies that systematically evaluate different stimulation intensities, duration, and electrode placement are lacking.

## Graphical Abstract

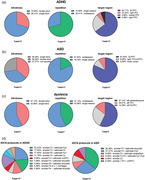

## Significant Outcomes

- In ADHD, prefrontal anodal tDCS has been more promising compared to the right IFG stimulation.
- In ASD and dyslexia, left prefrontal anodal tDCS and left temporoparietal anodal stimulation have been promising for improving behavioral and reading problems, respectively.
- Double‐blind, RCTs consisted of 70.5%, 36.3%, and 57.1% of tDCS studies in ADHD, ASD, and dyslexia, respectively, and tDCS was found safe with no reported serious side effects in 6587 sessions in 745 children and adolescents across 35 studies.

## Limitations

- Overall quality of established evidence in our systematic review was low, mostly because of small sample sizes, lack of long‐term follow‐ups, and several risks of bias.
- Heterogenous stimulation parameters in ADHD studies and low number of double‐blind RCTs in ASD are noticeable limitations in these disorders.

## 1 INTRODUCTION

Over the last two decades, transcranial direct current stimulation (tDCS) has been exponentially applied in humans for studying and modifying brain physiology that underlies cognition (Polanía et al., 2018; Salehinejad et al., 2021) as well as for improving symptoms in clinical populations that suffer from plasticity‐related symptoms/deficits (Fregni et al., 2020). Yet, the number of currently available studies in children and adolescents is limited compared to adults (Bikson et al., 2016). In the last couple of years, however, tDCS has been increasingly used in children and adolescents (Rivera‐Urbina et al., 2017; Salehinejad et al., 2021; Vicario & Nitsche, 2013, 2019). In child psychiatric disorders, tDCS has been mostly applied in neurodevelopmental disorders specifically attention‐deficit hyperactivity disorder (ADHD) (Salehinejad et al., 2021, 2016; Westwood et al., 2021), autism spectrum disorder (ASD) (García‐Gonz ález et al., 2021; Osório & Brunoni, 2019), and developmental dyslexia (Salehinejad et al., 2021; Turker & Hartwigsen, 2022).

What makes the application of tDCS, and other non‐invasive brain stimulation techniques, promising in these disorders is the underlying pathophysiology, which is related to brain functional and structural abnormalities. In ADHD pathophysiology, there are at least two influential theories that have gained support with neuroimaging, neuropsychological, and brain stimulation studies. The first theory posits that ADHD is a result of poor inhibitory control due to executive dysfunctions (Barkley, 1997; Willcutt et al., 2005), which are associated with functional abnormalities in the prefrontal cortex and several subcortical regions (Passarotti et al., 2010; Samea et al., 2019). The other theory, “motivational dysfunction theory” (Cepeda et al., 2000; Sonuga‐Barke, 2005) assumes that there are impulse control deficits that lead to hyperactivity, and these deficits are mostly related to the medial prefrontal regions and subcortical areas (Krain & Castellanos, 2006; Rubia, 2018). The most updated account on ADHD pathophysiology shows that it results from both *hot* and *cold* cognitive deficits that correspond to distinct but related brain regions although cold cognitive deficits seem to be central (Cubillo et al., 2012; Salehinejad et al., 2021). It is noteworthy that describing cognitive and executive functions as *hot* and *cold* is based on the extent they are related to emotion (e.g., hot) or purely cognitive aspects (e.g., cold) (Salehinejad et al., 2021; Ward, 2019).

In ASD and dyslexia, similar heterogeneous pathophysiology is documented. Impaired social cognition (e.g., theory of mind) and reciprocity behavior are core deficits in ASD (Lord et al., 2018). Neuroimaging studies have shown a frontal‐posterior network including the medial prefrontal cortex (e.g., ventromedial prefrontal cortex—vmPFC, posterior cingulate cortex, and bilateral temporoparietal junction—TPJ) and several subcortical regions (e.g., amygdala, insula, thalamus, and basal ganglia) with altered activation in ASD (Cerliani et al., 2015; Nijhof et al., 2018; Salehinejad et al., 2021; Yuk et al., 2020). A recent account of ASD pathophysiology posits that ASD is marked with both cognitive and social/emotional deficits related to cold and hot cognition but here hot cognition deficits seem more central (Salehinejad et al., 2021). Developmental dyslexia, as the most frequent learning disorder, is characterized by severe impairments in reading and writing despite normal intelligence (American Psychiatric Association 2013). Here, the left hemisphere and especially the frontal region (e.g., inferior frontal gyrus), temporal, parietal (e.g., inferior parietal regions), and also visual cortex and cerebellum are involved in language difficulties (D'mello & Gabrieli, 2018; Richlan et al., 2010).

In addition to brain functional abnormalities, these major neurodevelopmental disorders come with related cognitive, affective, and social deficits. Modulating cortical (and subcortical activities) with tDCS is assumed to regulate such functional abnormities and hopefully associated cognition and behavior. Cortical excitability and neuroplasticity are two fundamental physiological components underlying human cognition and behavior (Salehinejad et al., 2021), which can be modulated by tDCS (Polanía et al., 2018). Based on this assumption, tDCS has been applied for enhancing cognitive, emotional, and social functions in healthy individuals (Ghanavati et al., 2018; Ghanavati et al., 2019; Nejati et al., 2018; Sellaro et al., 2016) and also improving respective deficits in brain disorders (Begemann et al., 2020; Fregni et al., 2020; Vicario et al., 2019) including neurodevelopmental disorders.

Despite growing interest in the application of tDCS in neurodevelopmental disorders, the number of standard tDCS studies with robust experimental conditions is still limited and warrants further investigation. Furthermore, the standard and safe application of tDCS in the developing population, especially children and adolescents requires an updated overview of the currently available studies. Finally, results have been mixed regarding the efficacy of tDCS, especially in ADHD. The available reviews that are published in the last 2 years are mostly limited to one specific disorder, include studies with adult sample, or are relatively outdated. The only review with a similar scope was published in 2019 (Finisguerra et al., 2019) and includes 16 tDCS studies, 4 of which are case reports and/or open‐label trials with no risk of bias or safety evaluation for the included studies. Accordingly, here, we used the PRISMA (Preferred Reporting Items for Systematic Reviews and Meta‐Analyses) method to systematically review the latest reports of tDCS studies conducted to date in major neurodevelopmental disorders including ADHD, ASD, and dyslexia.

### 1.1 Aim of the study

We present a systematic review to (1) evaluate the efficacy of tDCS in improving the symptoms and neuropsychological deficits of these disorders and (2) investigate the safety aspects of tDCS in these pediatric populations. We also discuss future directions for tDCS studies in these disorders.

## 2 METHODS

### 2.1 Information sources, search strategy, and study selection

**FIGURE 1:**
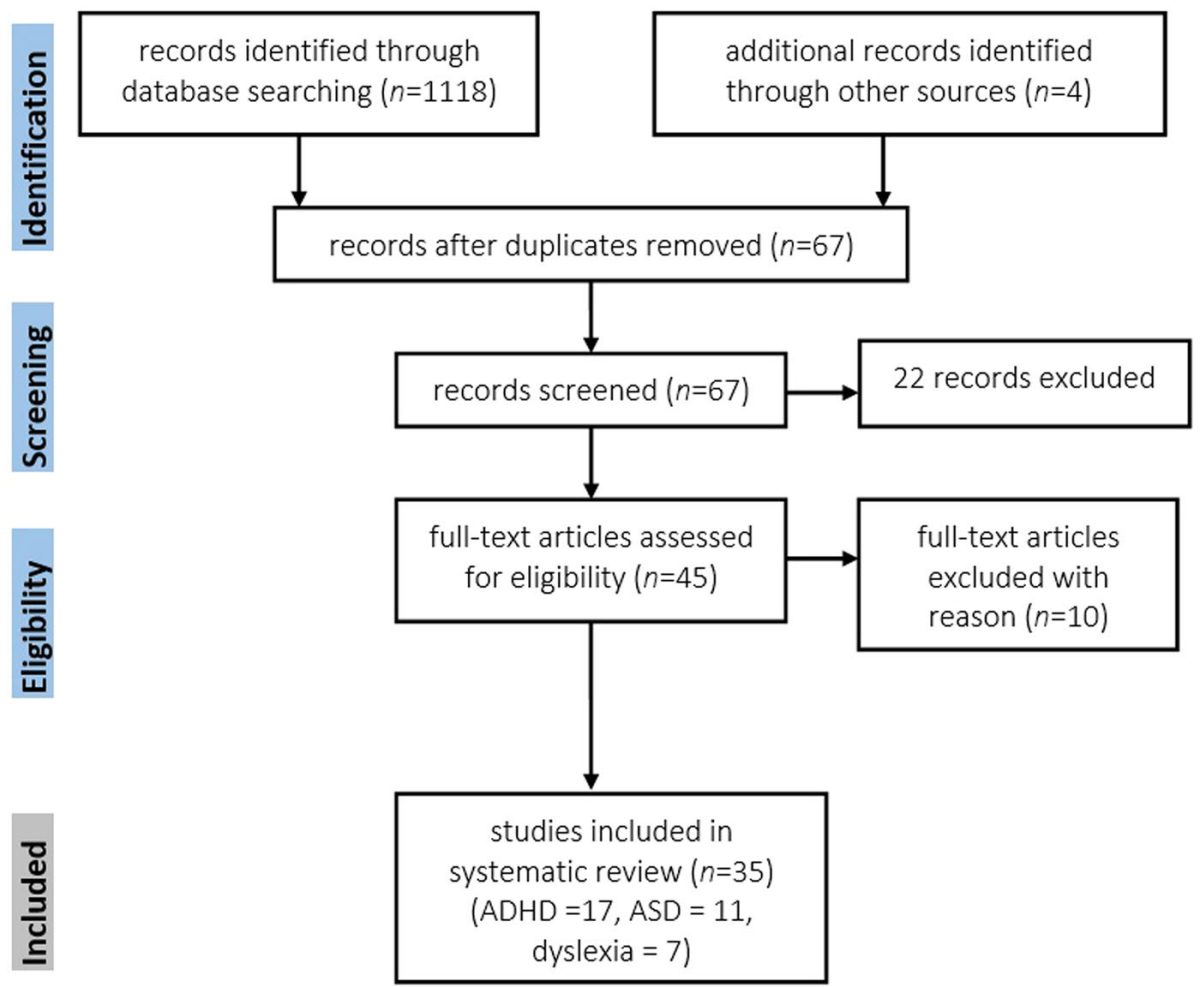
PRISMA flow diagram of included studies investigating the effects of transcranial direct current stimulation in ADHD, autism, and dyslexia. *Note*: Twenty‐two records were excluded for being conducted in adults, published as protocol, review articles, and case reports, and not meeting the inclusion criteria. Four non‐English full‐texts and non‐RCT (mainly open‐label trials) were excluded. Abbreviations: ADHD, attention‐deficit hyperactivity disorder; ASD, autism spectrum disorder

We used the PRISMA approach (Moher et al., 2015) in this systematic review and registered the protocol in PROSPERO (CRD42022321430). Using the PRISMA guidelines, a systematic search was performed by the first author in PubMed (Medline), Scopus, and Google Scholar, using the following search terms: [“ADHD” OR “attention‐deficit hyperactivity disorder” OR “ASD” OR “autism spectrum disorder” OR “dyslexia” OR “learning disorder” OR "lnaguage disorder"] AND [ “transcranial electrical stimulation” OR “transcranial direct current stimulation” OR “tES” OR “tDCS”] AND [“children” OR “pediatric” OR “adolescents”] with the final search updated on March 10, 2022. The database search identified initial 1118 records with 587 records identified via PubMed and 531 records identified via the Scopus database. After removing the duplicates of two databases, 67 records remained for screening. Furthermore, a manual search of the reference sections of the retrieved studies and review articles was carried out. No year limit was applied. Review articles, meta‐analyses, and relevant book chapters were examined for cross‐references. The PRISMA flow diagram is displayed in Figure 1.

### 2.2 Study inclusion

Only peer‐reviewed published studies were included in our analysis. The inclusion criteria were: (1) randomized controlled trials with placebo (sham)‐control, baseline‐control, or waitlist‐control, (2) studies published in international peer‐reviewed journals and in English, (3) studies conducted on children and adolescents with ADHD, ASD, or dyslexia (studies conducted on adults were excluded). The final search identified a total of 35 studies following screening 67 records. After removing duplicates and screening the abstracts based on the inclusion criteria, 17 RCTs in ADHD, 11 RCTs in ASD, and 7 RCTs in dyslexia remained for full‐text assessment and data extraction. It is of note that two recent tDCS studies in ADHD population belong to one dataset (Westwood et al., 2021, 2022) but as they are published separately with two different sample sizes and have different measures, we treated them as separate studies.

### 2.3 Risk of bias

**FIGURE 2:**
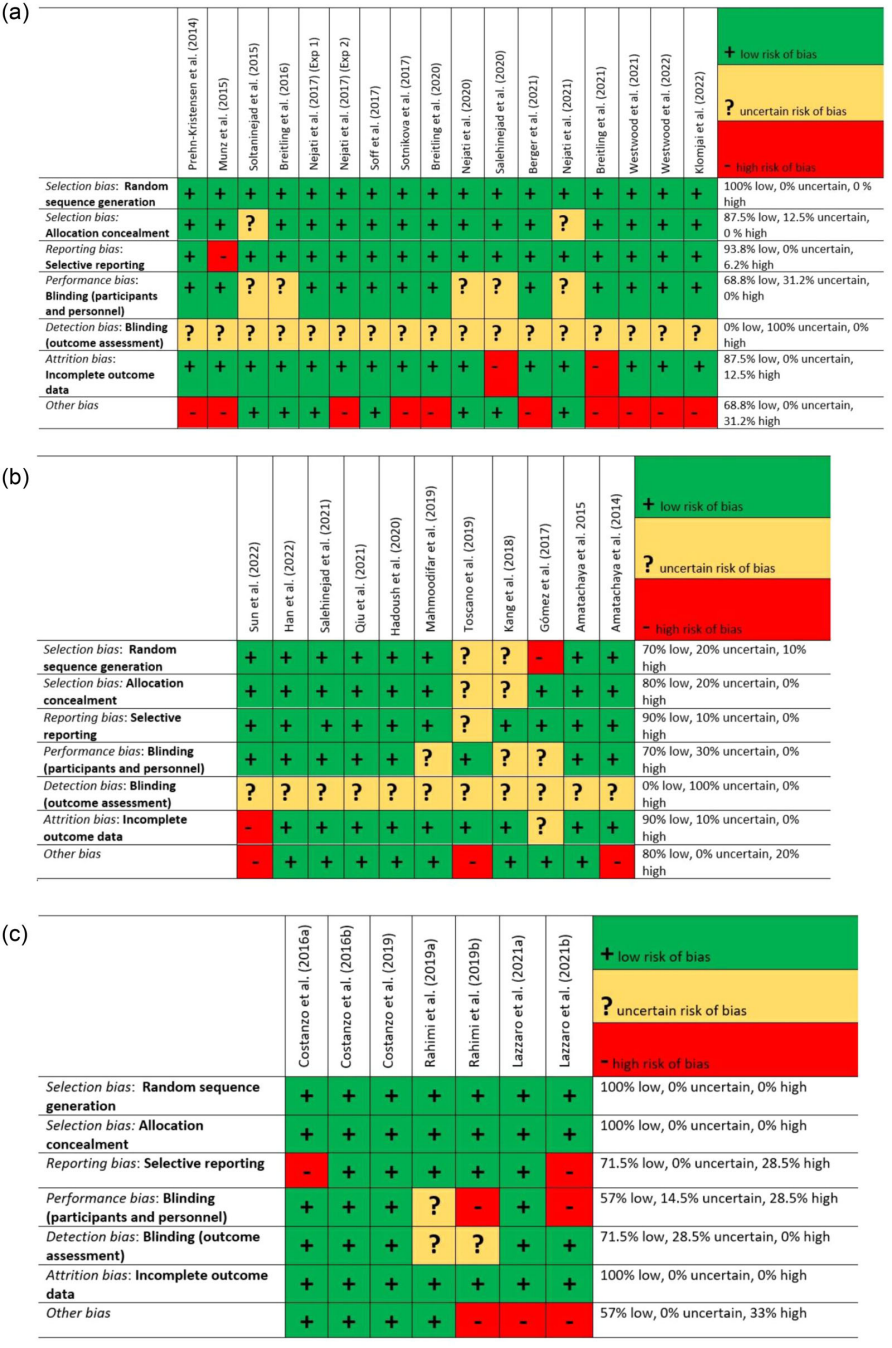
(a) Bias assessment for included tDCS studies in children and adolescents with ADHD (*n* = 17) using the Cochrane risk of bias tool. (b) Bias assessment for included tDCS studies in children and adolescents with ASD (*n* = 11) using the Cochrane risk of bias tool. (c) Bias assessment for included tDCS studies in children and adolescents with dyslexia (*n* = 7) using the Cochrane risk of bias tool; Abbreviations: na, not applicable

The risk of bias assessment was performed using the Cochrane Collaboration's tool (Higgins et al., 2011). In each study, authors judged the risk of selection, performance, detection, attrition, reporting, and other biases. The risk of bias was categorized as low, high, or uncertain, and a summary of results for tDCS studies conducted in ADHD, ASD, and dyslexia is shown in Figure 2.

## 3 RESULTS

### 3.1 Risk of bias

#### 3.1.1 tDCS studies in ADHD

The risk of bias for each tDCS study in ADHD is reported in Figure 2a. In ADHD studies, five studies used a single‐blind design (Breitling et al., 2016; Nejati et al., 2021; Nejati et al., 2020; Salehinejad et al., 2020; Soltaninejad et al., 2015) yielding a potential detection bias as the experimenter was not blind to the tDCS condition. We identified “other” biases in several other studies. In Breitling et al. (2020), the sources of other biases are different experimental procedures in the control and ADHD groups, and reduction of stimulation intensity to 50% in 3 out of 14 participants due to low tolerability of the standard current intensity. In Breitling‐Ziegler et al. (2021), the source of other bias includes low sample size in each dosage group (9 and 11) that is very low for concluding the efficacy of HD‐tDCS in ADHD. In Berger et al.’s (2021) study, the source of other bias is having no sham control condition although the authors had an active control condition. Finally, in Westwood and colleagues' recent studies (Westwood et al., 2021, 2022), the authors applied concurrent cognitive training and tDCS without any experimental condition that disentangles the effect of each intervention alone. In other marked studies, the source of other bias is related to low sample size (>15), which does not comply with current standards and guidelines for tES studies.

#### 3.1.2 tDCS studies in ASD

The risk of bias for each tDCS study in ASD is reported in Figure 2b. The source of *selection* bias in GÃmez et al.’s (2017) was that ASD children received different interventions (i.e., repetitive TMS for 11‐years‐old and older or tDCS in 10‐years‐old and younger) depending on age. Although both techniques are modulatory, their mechanisms of effect and focality and the modulated target regions are thus different. In Sun et al. (2022), the source of the other bias is related to comorbidity of patients. Most of the enrolled patients were accompanied by degrees of comorbidities including anxiety disorder, ADHD, and attenuated psychosis syndrome. The source of *other* biases in other studies (Amatachaya et al., 2014; Toscano et al., 2019) is having no control over medication use in the patients or comorbid disorders in addition to ASD, which makes the sample heterogenous.

#### 3.1.3 tDCS studies in dyslexia

The risk of bias for tDCS studies in children and adolescents with dyslexia is reported in Figure 2c. Four of 7 studies had a double‐blind design (Costanzo et al., 2019; Costanzo et al., 2016; Costanzo et al., 2016; Lazzaro et al., 2021) and 3 studies reported a single‐blind design (Lazzaro et al., 2021; Rahimi et al., 2019; Rahimi et al., 2019). In the multi‐session study conducted by Rahimi et al. (2019b), (there are two experimental groups one of which received the tDCS intervention. Furthermore, the control group was a waitlist group with not enough details about how this group was monitored. In the Lazzaro et al.’s (2021b) study (, there is no sham control condition and we considered this as an important other bias that does not allow us to rule out the potential placebo effect. Also, some participants in Lazzaro and colleagues' two studies (Lazzaro et al., 2021a,b) were taken from participants in Costanzo et al.’s (2019) study ), which might be a source of selection bias. The source of reporting bias in the Costanzo et al. (2016a) and Lazzaro et al. (2021b) studies is related to no report of reaction time in the n‐back test.

### 3.2 Overview of tDCS studies in children and adolescents with neurodevelopmental disorders

Of studies included in this review, ADHD is the most studied neurodevelopmental disorder with 17 tDCS RCTs in children and adolescents with ADHD. ASD is the second‐most studied neurodevelopmental disorder with 11 reported studies. Seven studies also reported the application of tDCS in developmental dyslexia. Details of the tDCS studies in these disorders are summarized in Tables 1, 2, 3, respectively. In what follows, we briefly overview the targeted outcome measures, study design, and important parameters of tDCS interventions applied in each disorder.

**TABLE 1:** TDCS studies in children and adolescents with attention‐deficit hyperactivity disorder (ADHD)—Latest update on March 2022

#	Author	Design (control condition)	*N*	Mean age ± SD [age range]	Target electrode site	Return electrode site/electrode size	Intensity	Duration	Polarity	Outcome measure	Major finding
1	Prehn‐Kristensen et al. (2014)	RCT double blind (sham controlled)	12	12 ± 1.4, [10−14]	Left dlPFC (F3)/right dlPFC (F4)	Lateral mastoid 0.503 cm2 (Ag/AgCl electrodes)	(0−250 μA 0.75 Hz)	5 × 5 min (2 single sessions)	Anodal	Declarative memory	Enhanced memory consolidation and retrieval following active tDCS vs sham tDCS
2	Munz et al. (2015)	RCT double blind (sham controlled)	14	12.3 ± 1.39, [10−14]	Left dlPFC (F3)/right dlPFC (F4)	Lateral mastoid 0.503 cm2 (Ag/AgCl electrodes)	(0−250 μA 0.75 Hz)	5 × 5 min (2 single sessions)	Anodal	Response inhibition	Faster response time after active vs sham tDCS in Go/No‐Go task. No effect on accuracy
3	Soltaninejad et al. (2015)	RCT single blind (sham controlled)	20	16.40 ± 1.09 [15−17]	Left dlPFC (F3)	Right supraorbital (Fp2)/7 × 5 cm	1.5 mA	15 min (3 single sessions)	Anodal/cathodal	Response inhibition, selective attention	Cathodal F3, but not anodal F3, improved response inhibition. No effect on selective attention
4	Breitling et al. (2016)	RCT parallel‐group single blind (sham controlled)	21	14.33 [NR]	Right IFG (F8)	Left mastoid	1 mA	20 min (3 single sessions)	Anodal/cathodal	Response inhibition, interference control	No effect on interference control after anodal/cathodal tDCS, diminished commission errors in the ADHD group vs healthy controls after anodal tDCS
5	Nejati et al. (2017) (Exp 1)	RCT double blind (sham controlled)	15	10 ± 2.3 [8–15]	Bilateral dlPFC (anodal left)	Right dlPFC (F4)/5 × 5 cm	1 mA	15 min (2 single sessions)	Anodal	Response inhibition, working memory, executive functions	Improved executive control functions (working memory, interference control) but not response inhibition and cognitive flexibility after active vs sham
6	Nejati et al. (2017) (Exp 2)	RCT double blind (sham controlled)	10	9 ± 1.8 [7–12]	Left dlPFC (F3)	Right supraorbital (Fp2)/5 × 5 cm	1 mA	15 min (3 single sessions)	Anodal/cathodal	Response inhibition, working memory, cognitive flexibility	Improved working memory after anodal tDCS over F3, improved response inhibition after cathodal tDCS over F3, improved cognitive flexibility after both protocols vs sham
7	Soff et al. (2017)	RCT double blind (sham controlled)	15	14.20 ± 1.2 [12−16]	Left dlPFC (F3)	Vertex (Cz)/7 × 5 cm	1 mA	10 × 20 min (daily) [5 active + 5 sham]	Anodal	ADHD symptoms	Reduced inattention and hyperactivity symptoms in the active tDCS vs sham condition
8	Sotnikova et al. (2017)	RCT double blind (sham controlled)	13	14.33 ± 1.2 [12−16]	Left dlPFC (F3)	Vertex (Cz)/7 × 5 cm	1 mA	20 min (2 single sessions)	Anodal	Quantified Behavior Test	Reduced RT and variability, reduced accuracy and increased omission errors increased connectivity in left DLPFC in the active tDCS vs sham
9	Breitling et al. (2020)	RCT double blind (sham controlled)	14	13.3 ± 1.9 [10–16]	Right IFG (F8)	Fp1/7 × 5 cm 1 cm electrodes (HD)	1 mA, 0.5 mA (4×1 montage)	20 min (3 single sessions)	Anodal	2‐back working memory task, task‐based EEG	No effect of conventional or HD‐tDCS on working memory. Higher responder rate for 4×1 (50%) than conventional (35%) tDCS. Higher N200 and P300 amplitudes after both protocols
10	Nejati et al. (2020)	RCT single blind (sham controlled)	20	8.60 ± 1.56	Left dlPFC (F3)/right vmPFC	Right vmPFC/left dlPFC (F3)/6 × 4 cm	1 mA	15 min (3 single sessions)	Anodal/cathodal	Reward processing, risky decision making	Anodal right vmPFC‐cathodal left DLPFC reduced risky decision‐making and delay discounting
11	Salehinejad et al. (2020)	RCT single blind (sham controlled)	17	9.33 ± 1.50	Right posterior parietal cortex (P4)	Left shoulder/7 × 5 cm	1 mA	15 min (2 single sessions)	Anodal	Attentional functioning	Anodal r‐PPC tDCS specifically improved attention orienting network but had a deteriorating effect on the top‐down attentional control
12	Berger et al. (2021)^a^	RCT double blind (no sham control)	19	13.3 ± 1.9 [7–12]	Left dlPFC (F3)	Right supraorbital (Fp2)/5 × 5 cm	0.75 mA	10 × 20 min (daily) [5 tDCS, 5 tRNS]	Anodal	ADHD symptoms, working memory, attentional performance	Bilateral dlPFC tRNS with cognitive training reduced ADHD rating‐scale score and working memory from baseline compared to tDCS. tRNS effects were larger than tDCS.
13	Nejati et al. (2021)	RCT single blind (sham controlled)	24	9.25 ± 1.53	Right dlPFC (F4)	Left shoulder/5 × 5 cm	1 mA	20 min (2 single sessions)	Anodal	Attention, response inhibition	Anodal tDCS of right dlPFC enhanced response inhibition in the circle tracing task and flanker incongruent trials but not Stroop and Go/No‐Go task performance. No‐Go response improved in children with mild symptom severity
14	Breitling et al. (2021)	RCT double blind (sham controlled)	33	13.3 ± 1.9 [10–17]	Right IFG (F8)	(4×1 montage)/1 cm electrodes (HD)	0.5 (*n* = 9) or 0.25 mA (*n* = 11)	5 × 20 min (daily)	Anodal	Working memory, response inhibition, task‐based EEG	0.25 mA increased commission error while 0.5 mA improved attention even 4 months after the stimulation. Distinct effects of tDCS with different current intensities.
15	Westwood et al. (2021)^a^, ^b^	RCT double blind (sham controlled)	50	13.3 ± 1.9 [10–18]	Right inferior frontal cortex (F8)	Left supraorbital (Fp1)/5 × 5 cm	1 mA	15 × 20 min (daily)	Anodal + cognitive training	ADHD symptoms, response inhibition, attention	ADHD rating scales were significantly lower at post‐treatment after sham relative to anodal tDCS. No other effects were significant. rIFC tDCS combined with cognitive training may not be
16	Westwood et al. (2022)^a^, ^b^	RCT double blind (sham controlled)	23	164.18 ± 227^c^ [10–18]	Right inferior frontal cortex (F8)	Left supraorbital (Fp1)/5 × 5 cm	1 mA	15 × 20 min (daily)	Anodal + cognitive training	EEG power, ERP	No significant sham versus anodal tDCS group differences in QEEG spectral power during rest and Go/No‐Go task performance
17	Klomjai et al. (2022)	RCT double blind crossover (sham controlled)	11	8.55 ± 0.65 [7–14]	Left dlPFC (F3)	Right supraorbital (Fp2)/5 × 5 cm	1.5 mA	5 × 15 min (daily)	Cathodal	Resting EEG, response inhibition, attention	After five active sessions, alpha and delta power increased in the right and left frontal areas. Omission errors decreased during go/no‐go tasks, with no differences at follow‐ups. No effect on attention

*Note*: tDCS = transcranial direct current stimulation; tRNS = transcranial random noise stimulation; RCT = randomized controlled trial; SD = standard deviation; RT = reaction time; dlPFC = dorsolateral prefrontal cortex; vmPFC = ventromedial prefrontal cortex; IFG = inferior frontal gyrus; rIFC = right inferior frontal cortex; Cz = vertex; F3 = left dorsolateral prefrontal cortex; F4 = right dorsolateral prefrontal cortex; P4 = right posterior parietal cortex; Fp1 = left supraorbital area; Fp2 = right supraorbital area; F8 = right inferior frontal gyrus; ERP = event‐related potential; NR = not reported or available.

^a^ Patients in these studies underwent tDCS intervention + cognitive training.

^b^ Age of patients in this study is reported in months.

**TABLE 2:** TDCS studies in children and adolescents with autism spectrum disorder—Latest update on March 2022

#	Author	Design (control condition)	*N*	Mean age ± SD [age range]	Target electrode site	Return electrode site/electrode size	Intensity	Duration	Polarity	Outcome measure	Major finding
1	Amatachaya et al. (2014)	RCT double blind (sham controlled)	20	6.4 ± 1.1 [5–8]	Left dlPFC (F3)	Right shoulder/7 × 5 cm	1 mA	10 × 20 min (daily) [5 active + 5 sham]	Anodal	Symptoms (psychosocial, cognition)	Anodal F3 tDCS vs sham tDCS, improved social function, behavioral, sensory/cognitive, ATEC scores)
2	Amatachaya et al. (2015)	RCT double blind (sham controlled)	20	6.4 ± 1.1 [5–8]	Left dlPFC (F3)	Right shoulder/7 × 5 cm	1 mA	20 min (single session)	Anodal	Symptoms (psychosocial, cognition), EEG correlates	Improved social behavior and behavioral ATEC scores after active tDCS associated with increased alpha frequency
3	Gómez et al. (2017)	RCT single blind (sham controlled)	24	12.2 [NR]	Left dlPFC (F3)	Right arm/NR	1 mA	20 × 20 min (daily)	Cathodal	Connectivity, ERP components, behavioral and social functioning	Increased functional connectivity. Shorter P300 latency, but no change in amplitude. Behavioral and social improvement for up to 6 months
4	Kang et al. (2018)	RCT wait‐list control trial	26	6.4 ± 1.7 [4–8]	Left dlPFC (F3)	Right supraorbital (Fp2)/7 × 4.5 cm	1 mA	10 × 20 min (every 2 days)	Anodal	EEG complexity with maximum entropy ratio (MER)	MER value significantly increased after tDCS in the experimental group
5	Toscano et al. (2019)^a^	RCT NR (sham controlled)	16	NR [9–14]	Left dlPFC (F3)	Right cerebellum/NR	1 mA age < 10 1.5 mA age > 11	20 × 20 min (daily)	Anodal	Behavioral symptoms, treatment evaluation	Significant decrease in the behavior and treatment evaluation checklist in the active tDCS vs sham condition
5	Mahmoodifar & Sotoodeh (2020)	RCT NR (sham controlled)	18	10.17 ± 2.75 [6–14]	Left motor cortex (M1)	Right supraorbital (Fp2)/7 × 5 cm	1.5 mA	10 × 20 min + motor training	Anodal	Motor skill learning, movement balance	Both anodal/sham tDCS combined with motor training improved balance. Active tDCS+training showed a significantly higher improvement compared to sham+training
7	Hadoush et al. (2020)	RCT double blind (sham controlled)	43 [healthy control *n* = 22]	7.8 ± 2.5 [4–15]	Left and right frontocentral (FC1‐FC2)	Left and right supraorbital (Fp1‐Fp2)/8 cm2	1 mA per electrode	10 × 20 min (daily)	Bilateral anodal	Symptoms (by ATEC)	Bilateral anodal tDCS significantly improved sociability, behavior, health, and physical conditions measured by ATEC with no reported side effects
8	Salehinejad et al. (2021)	RCT single blind (sham controlled)	14	10.7 ± 1.9	Right temporoparietal junction (CP6) vmPFC (Fpz)	Left shoulder/5 × 5 cm	1 mA	20 min (3 single sessions)	Anodal	Theory of Mind Test	Anodal vmPFC tDCS significantly improved ToM in children with ASD compared with both, rTPJ tDCS, and sham stimulation
9	Qiu et al. (2021)	RCT single blind (sham controlled)	40	NR [2–6]	Left dlPFC (F3)	Right shoulder/5 × 5 cm	1 mA	15 × 20 min (daily)	Anodal	Symptoms (by CARS and ABC), sleep habits	Real tDCS, but not sham tDCS significantly reduced the scores of CARS and sleep habits but not ABC scores
10	Han et al. (2022)	RCT double blind (sham controlled)	41	17.06 ± 2.45 [6–17]	Left dlPFC (F3)	Right supraorbital (Fp2)/5 × 5 cm	1 mA	10 × 20 min (daily)	Anodal + cognitive training	Social functioning, Hot and cold EFs, fNIRS functional connectivity	Multi sessions anodal left dlPFC tDCS + cognitive training improved social functioning, cognitive flexibility and functional connectivity of the right medial PFC
11	Sun et al. (2022)^b^	RCT single blind parallel group (sham controlled)	37	7.80 ± 2.00 [NR]	Left dlPFC (F3)	Right supraorbital (Fp2)/7 × 5 cm	1.5 mA	12 × 20 min (daily)	Anodal + rehabilitation	Symptoms (by ABC), ERP (MMN)	ABC significantly improved n after active and sham tDCS+ rehabilitation. Active tDCS group performed significantly better. MMN amplitude increased in both groups with no significant difference between groups

*Note*: tDCS = transcranial direct current stimulation; RCT = randomized controlled trial; SD = standard deviation; ERP = event‐related potential; dlPFC = dorsolateral prefrontal cortex; vmPFC/Fpz = ventromedial prefrontal cortex; F3 = left dorsolateral prefrontal cortex; F4 = right dorsolateral prefrontal cortex; Fp1 = left supraorbital area; Fp2 = right supraorbital area; M1 = left primary motor cortex; CP6/rTPJ = right temporoparietal junction; FC1/FC2 = left and right frontocentral regions; ATEC = Autism Treatment Evaluation Checklist; CARS = Childhood Autism Rating Scale; ABC = Aberrant Behavior Checklist; MMN = mismatch negativity; NR = not reported or available.

^a^ Findings of these works are based on proceeding reports.

^b^ Patients in these studies underwent tDCS intervention + rehabilitation treatment.

**TABLE 3:** TDCS studies in children and adolescents with dyslexia—Latest update on March 2022

#	Author	Design (control condition)	*N*	Mean age ± SD [age range]	Target electrode site	Return electrode site/electrode size	Intensity	Duration	Polarity	Outcome measure	Major finding
1	Costanzo et al. (2016a)	RCT double blind (sham controlled)	19	13.7 ± 2.4 [10–17]	Left parietotemporal (mid P7‐TP7)	Right parietotemporal/5 × 5 cm	1 mA	20 min (3 single sessions)	Anodal/cathodal	Reading abilities	Anodal left cathodal right temporoparietal tDCS improved reading accuracy. The reverse protocol decreased accuracy
2	Costanzo et al. (2016b)	RCT double blind (sham controlled)	18	13.2 ± 2.6 [10–17]	Left parietotemporal (mid P7‐TP7)	Right parietotemporal/5 × 5 cm	1 mA	18 × 20 min	Anodal	Reading abilities	Reduced reading errors and increased reading speed after active tDCS vs sham up to 1 month
3	Costanzo et al. (2019)	RCT double blind (sham controlled)	26	13.6 ± 2.4 [10–17]	Left parietotemporal (mid P7‐TP7)	Right parietotemporal/5 × 5 cm	1 mA	18 × 20 min	Anodal	Reading abilities	Improved non‐word and low frequency word reading after active tDCS vs sham up to 6 months
4	Rahimi et al. (2019a)	RCT single blind (sham controlled)	17	10.35 ± 1.36 [9–12]	Bilateral STG (T7,T8) Left STG (T3,T4)	Right shoulder/5 × 5 cm	1 mA	20 min (3 single sessions)	Anodal	Auditory processing and ERP correlates	Improved visual attention processing in active tDCS vs sham
5	Rahimi et al. (2019b)	RCT single blind (waitlist control)	45 (tDCS group = 15)	Primary school age 2–5 grade [7–10]	Left dlPFC (F3)	NR/5 × 5 cm	1.5 mA	10 × 20 min (daily)	Anodal	Visual sustained attention	Left dlPFC tDCS Improved visual attention processing in active tDCS vs sham in children with a specific learning disorder
6	Lazzaro et al. (2021a)	RCT double blind (sham controlled)	26	13.80 ± 2.3 [10.8–17.8]	Left TPJ (between P7‐TP7)	Right TPJ (between P8‐TP8)/5 × 5 cm	1 mA	18 × 20 min	Anodal/cathodal	Word and pseudoword reading	Anodal left cathodal right TPJ tDCS in the active group improved word reading fluency in dyslexia
7	Lazzaro et al. (2021b)	RCT single blind (no sham)	10	13.89 ± 2.4 [10.8–16.7]	Left TPJ (between P7‐TP7)	Right TPJ (between P8‐TP8)/5 × 5 cm	1 mA	20 min (single session)	Anodal/cathodal	Word and pseudoword reading	Anodal left cathodal right TPJ improved text reading accuracy, word recognition, speed, motion perception, and modified attentional focusing

*Note*: tDCS = transcranial direct current stimulation; RCT = randomized controlled trial; SD = standard deviation; ERP = event‐related potentials; dlPFC = dorsolateral prefrontal cortex; F3 = left dorsolateral prefrontal cortex; T3/T4 = left and right temporal cortex; P7 = left parietal‐temporal region; TP7 = left parietal‐central region; P8 = right parietal‐temporal region; TP8 = right parietal‐central region; STG = superior temporal gyrus; TPJ = temporoparietal junction.

#### 3.2.1 ADHD

We found 17 tDCS studies in children and adolescents with ADHD (Berger et al., 2021; Breitling et al., 2016; Breitling et al., 2020; Breitling‐Ziegler et al., 2021; Klomjai et al., 2022; Munz et al., 2015; Nejati et al., 2021; Nejati et al., 2020; Nejati et al., 2017; Prehn‐Kristensen et al., 2014; Salehinejad et al., 2020; Soff et al., 2017; Soltaninejad et al., 2015; Sotnikova et al., 2017; Westwood et al., 2022; Westwood et al., 2021). It is of note that the 2 studies of Westwood and colleagues are from the same database, but as they report different measures with different sample sizes, we listed them separately. Cognitive deficits and executive dysfunctions were the primary targets in 10 studies. In other studies, one specifically targeted behavioral symptoms (Soff et al., 2017), 2 studies investigated both cognitive deficits and symptoms improvement (Berger et al., 2021; Westwood et al., 2021), and 4 studies also examined EEG power spectral and task‐based EEG in addition to cognitive deficits (Breitling et al., 2020; Breitling‐Ziegler et al., 2021; Klomjai et al., 2022; Westwood et al., 2022). Details of these studies including stimulation protocols, sample size, outcome measures, and major findings are summarized in Table 1. Overall, the results of these studies suggest partially improving effects of tDCS on cognitive deficits (response inhibition, working memory, attention, cognitive flexibility, reward processing), but the clinical utility of tDCS in ADHD cannot yet be concluded and requires further investigation with multi‐session protocols in larger sample sizes (Salehinejad et al., 2019; Salehinejad et al., 2020). Of 5 studies with multi‐session protocols (Berger et al., 2021; Breitling‐Ziegler et al., 2021; Klomjai et al., 2022; Soff et al., 2017; Westwood et al., 2022; Westwood et al., 2021), 40% reported a significant improving effect on outcome variables including ratings of symptoms and one study (2021) found beneficial effect of 5 day HD‐tDCS on attention (but not response inhibition) which was detectable up to 4 months after the stimulation.

**FIGURE 3:**
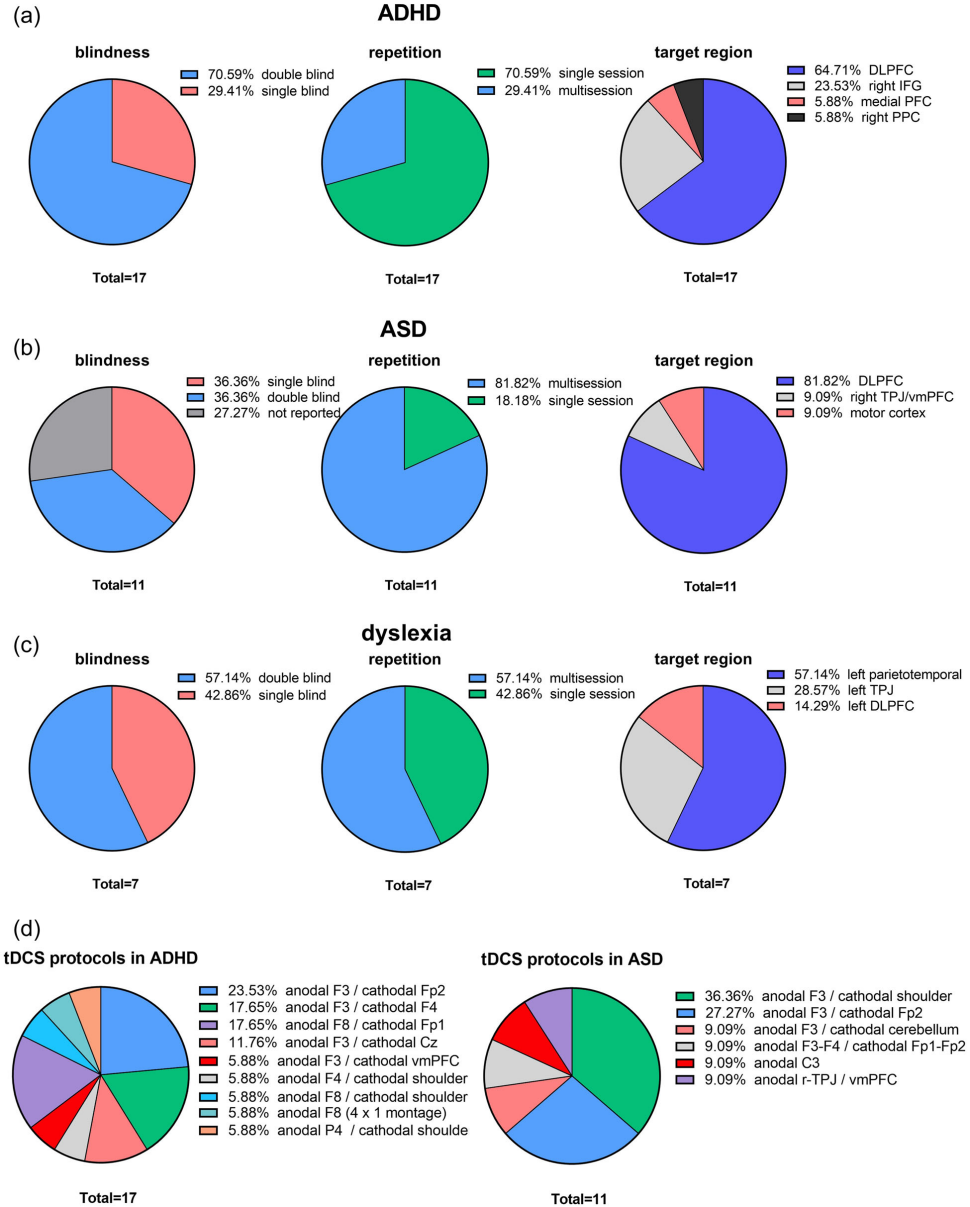
(a) Proportion of study blindness, repetition (single vs. multi session), and target regions of tDCS studies in children and adolescents with ADHD (*n* = 17). (b) Proportion of study blindness, repetition (single vs. multi session), and target regions of tDCS studies in children and adolescents with ASD (*n* = 11). (c) Proportion of study blindness, repetition (single vs. multi session), and target regions of tDCS studies in children and adolescents with dyslexia (*n* = 7). (d) Proportion of stimulation protocles of tDCS studies in ADHD (*N* = 17) and ASD (*N* = 11). Abbreviations: ADHD, attention‐deficit hyperactivity disorder; ASD, autism spectrum disorder; DLPFC, dorsolateral prefrontal cortex; IFG, inferior frontal gyrus; PFC, prefrontal cortex; PPC, posterior parietal cortex; TPJ, right temperoparital junction; vmPFC, ventromedial prefrontal cortex; F3/F4, left and right DLPFC; Fp1/Fp2, left and right supraorbital area; F8, right IFG; Cz, vertex; P4, right PPC; C3, left motor area

The left dlPFC was the most often targeted region, and anodal tDCS—the most often applied protocol—with promising results (Figure 3a,d). Additional cortical regions such as the medial prefrontal cortex, right inferior frontal gyrus, and right dlPFC are also involved in the pathophysiology of ADHD, which were not explored in studies published before 2020. Recently, however, one study targeted the medial prefrontal cortex (e.g., vmPFC) and found that tDCS over this region vs. the left dlPFC improved hot executive dysfunction (e.g., risky decision‐making and delay discounting) in ADHD (Nejati et al., 2020). Another study also found that tDCS over the right posterior parietal cortex had a partial and specific effect on attentional orienting but not attentional alerting or attentional control and, on the contrary, had a deteriorating effect on the top‐down attentional control (Salehinejad et al., 2020). The specific role of the right dlPFC with anodal tDCS has been studied in one study so far (Nejati et al., 2021) where the reference electrode was placed externally. With four published tDCS studies in 2020–2022, the number of studies that targeted r‐IFG in children and adolescents with ADHD is now five (Breitling et al., 2016; Breitling et al., 2020; Breitling‐Ziegler et al., 2021; Westwood et al., 2022; Westwood et al., 2021), which may allow us to understand the contribution of this region. Overall, these studies found no significant improving effect of r‐IFG anodal tDCS on their primary outcome measures including working memory, response inhibition, ADHD symptoms, or EEG markers. In two studies, however, (Breitling et al., 2016; Breitling‐Ziegler et al., 2021), significant improvement was reported in reducing commission errors and improving attention which was detectable up to 4 months after the end of stimulation. The results of these studies need to be interpreted with some considerations about the applied protocols and experimental procedure, which we explain in the discussion.

It is also of note that only 2 studies applied HD‐tDCS protocols in which usually 4 return electrodes surround on a central electrode (Breitling et al., 2020; Breitling‐Ziegler et al., 2021) and the rest applied conventional tDCS protocols with electrode sizes of 25 or 35 cm. Anodal polarity was the predominant target in 16 studies and in 4 studies both anodal and cathodal polarity were applied (Breitling et al., 2016; Nejati et al., 2020; Nejati et al., 2017; Soltaninejad et al., 2015). Only in 3 of these studies (Klomjai et al., 2022; Nejati et al., 2017; Soltaninejad et al., 2015), cathodal tDCS was applied over the left dlPFC, which improved outcome variables (response inhibition). Finally, only 5 studies applied the return electrode extracranially on either mastoid or shoulders (Breitling et al., 2016; Munz et al., 2015; Nejati et al., 2021; Prehn‐Kristensen et al., 2014; Salehinejad et al., 2020). The stimulation intensities also ranged from 0.25 to 0.5 mA for HD‐tDCS, and 0.75 and 1.5 mA for conventional tDCS protocols. See Table 1 for specific study details and Figure 3a for an overview of studies blindness, repetition rate, and target regions.

#### 3.2.2 Autism spectrum disorder

We identified 11 RCTs of tDCS application in children and adolescents with ASD. Details of these studies including stimulation protocols, sample size, outcome measures, and major findings are summarized in Table 2. DLPFC was the most often‐targeted region (Figure 3b,d) and here, anodal tDCS over the left dlPFC (in 7 out of 11 studies) (Amatachaya et al., 2014; Amatachaya et al., 2015; Han et al., 2022; Kang et al., 2018; Qiu et al., 2021; Sun et al., 2022; Toscano et al., 2019) was the most often applied protocol in ASD. In one study (Hadoush et al., 2020), a significant improving effect of bilateral anodal dlPFC tDCS was observed on overall behavioral symptoms. A novel aspect of this study was the application of bilateral anodal stimulation over both, left and right dlPFC, with a 4×1 electrode arrangement. Cathodal stimulation over the left dlPFC is also applied in ASD (GÃmez et al., 2017) and in two open‐label studies that were not included in this review. Two recent studies also reported improving effects of frontocerebellar tDCS (i.e., anodal left dlPFC, cathodal right cerebellar tDCS) on behavioral symptoms (Toscano et al., 2019), and anodal stimulation over the primary motor cortex on motor skill training in children with ASD (Mahmoodifar & Sotoodeh, 2020). Of 11 included studies, only 1 study targeted right TPJ and vmPFC, two key regions in the theory of mind in ASD, and found that anodal vmPFC, but not r‐TPJ tDCS, significantly improved ToM in children with ASD (Salehinejad et al., 2021).

Nine of 11 studies had repeated protocols in which tDCS was applied on consecutive days for 10, 12, 15, or 20 sessions (Amatachaya et al., 2014; GÃmez et al., 2017; Hadoush et al., 2020; Han et al., 2022; Kang et al., 2018; Mahmoodifar & Sotoodeh, 2020; Qiu et al., 2021; Sun et al., 2022; Toscano et al., 2019). All these studies reported significant improvement of their outcome variables (mostly ASD symptoms) and repeated daily sessions resulted in improved behavioral and social functioning for up to 6 months (GÃmez et al., 2017) after the intervention. The stimulation intensity varied from 1 mA (*n* = 9) to 1.5 mA (*n* = 2) and all studies used conventional protocols with electrode sizes of 25 or 35 cm. Only 4 studies applied a double‐blind design (Amatachaya et al., 2014; Amatachaya et al., 2015; Hadoush et al., 2020; Han et al., 2022) and the rest had a single‐blind design or not reported the study blinding (Figure 3b), which should be considered with care. See Table 2 for major findings of the studies and Figure 3b for an overview of studies blindness, repetition rate, and the target regions.

#### 3.2.3 Dyslexia

Seven tDCS RCTs in developmental dyslexia (Costanzo et al., 2019; Costanzo et al., 2016; Costanzo et al., 2016; Lazzaro et al., 2021; Lazzaro et al., 2021; Rahimi et al., 2019; Rahimi et al., 2019) were included in this review. Reading performance and abilities were primary outcome measures in 5 of 7 studies and in all of them, a significant improvement was observed in reading components (e.g., reading accuracy, word frequency, reading speed, reading fluency). One study examined sustained attention in children with dyslexia (Rahimi et al., 2019) and in another study, the outcome measure was auditory processing and its ERP correlates (Rahimi et al., 2019), which showed improved auditory processing. Unlike tDCS studies in ADHD and ASD, temporoparietal regions (e.g., temporoparietal junction, superior temporal gyrus, P7/8, TP7/8, T3, T4 according to the 10/20 EEG international system) were the target regions in all of the studies except one that targeted left dlPFC for improving sustained attention in children with dyslexia (Rahimi et al., 2019).

Stimulation intensity ranged from 1 mA (*n* = 6) to 1.5 mA (*n* = 1) and anodal polarity was predominant in all studies, especially on the left hemisphere. In 71.4% of studies (5 of 7), bilateral tDCS was applied with anodal left and cathodal right parietotemporal regions (Costanzo et al., 2019; Costanzo et al., 2016; Costanzo et al., 2016; Lazzaro et al., 2021; Lazzaro et al., 2021). In 4 studies, repeated tDCS sessions (10 or 18 sessions) were applied and all of them reported significantly improved reading abilities (Costanzo et al., 2019; Costanzo et al., 2016; Lazzaro et al., 2021) or visual sustained attention (Rahimi et al., 2019) in children and adolescents with dyslexia. Details of these studies including stimulation protocols, sample size, outcome measures, and major findings are summarized in Table 3. See also Figure 3c for an overview of studies blindness, repetition rate, and the target regions.

### 3.3 Safety

In the included studies (*n* = 35), a total of 6587 sessions of tDCS were conducted in 745 children and adolescents with ADHD, ASD, or dyslexia, and no serious adverse effect was reported during or after the tDCS. In the majority of studies, reported side effects were limited to skin sensations (e.g., itching, tingling, or mild burning) which were transient. Several unexpected mild occurrences were reported though. In ADHD studies, one study reported one case of headache after anodal tDCS (Soff et al., 2017). In another study (Sotnikova et al., 2017), one participant felt nervous or overexcited during stimulation and another reported headache. In one study that investigated blinding successfulness based on reported side effects (Breitling‐Ziegler et al., 2021), the intensity of painful sensation was rated on average as 0.94 on a six‐point Likert scale and 86% of individuals were willing to participate again in a tDCS study. No serious or unusual side effects were reported in ASD tDCS studies. Similarly, in tDCS studies conducted on dyslexia, side effects were limited to mild tingling, itching, and burning and no participants withdrew from the study due to discomfort.

## 4 DISCUSSION

In this systematic review, we investigated efficacy and safety of the randomized‐controlled trials that applied tDCS in 3 major neurodevelopmental disorders: ADHD, ASD, and dyslexia. With regard to efficacy and regardless of effect size, tDCS was found at least partially effective in 100% of the studies conducted in children and adolescents with ASD (*n* = 11) and dyslexia (*n* = 7) (see Tables 2 and 3 last columns). In 64.7% of tDCS studies in children with ADHD (*n* = 11 of 17), a significant improving effect on at least one of the outcome variables was observed. Moreover, 88.8% (16 of 18) of all multi‐session tDCS protocols applied in 18 studies (ADHD = 5, ASD = 9, dyslexia = 4) reported significant improvement in their outcome variables (3 of 5 studies in ADHD, 9 of 9 studies in ASD, and 4 of 4 studies in dyslexia), including clinical symptoms in 8 studies (ADHD = 1, ASD = 7). These results are overall promising, especially for ASD and dyslexia, yet cannot establish clinical efficacy of tDCS unless proved in large clinical trials with robust experimental design. Indeed, analyzing the effect size in previous metanalyses has shown small effect or trend‐level improvements of tDCS in ADHD (Salehinejad et al., 2019; Westwood et al., 2021), which is partly due to heterogeneity in stimulation protocols and outcome measures. Assessment of biases of the included studies shows that there is a need for randomized clinical trials with a double‐blind design in all 3 groups, especially ASD. With regard to safety, no single report of serious adverse effects was reported in these 35 studies confirming the safety of tDCS in children and adolescents in line with recent studies (Bikson et al., 2016; Salehinejad et al., 2021; Zewdie et al., 2020). In what follows, we discuss important methodological considerations for each disorder that are noteworthy.

### 4.1 ADHD

Two brain regions were targeted in the majority of RCTs in ADHD: the lateral prefrontal cortex and the r‐IFG. The dlPFC, specifically left dlPFC, is the most‐often targeted region, which is not surprising due to its documented role in executive functions (Koechlin et al., 2003; Miller & Cohen, 2001; Salehinejad et al., 2021). The right dlPFC, however, is not sufficiently investigated in ADHD tDCS studies. Right prefrontal regions especially the right IFG and dlPFC are well documented in response inhibition (Aron et al., 2014, Aron et al., 2004). The only tDCS study that specifically investigated the role of right dlPFC found a partial improving effect of right dlPFC tDCS (single session) in response inhibition, which was dependent on symptoms severity (Nejati et al., 2021). Future studies should investigate contribution of this region to ADHD cognitive deficits and symptoms with multi‐session experimental design and optimized protocol parameters. For example, it is still not known which stimulation protocol for right DLPFC (e.g., anodal/cathodal unilateral, anodal/cathodal bilateral dLPFC, anodal/cathodal right DLPFC with other regions) is more beneficial to ADHD psycho‐ and‐ neuropathology. Recently, we applied a single session of anodal tDCS over both left and right dlPFCs and found no effects on executive functions (2022).

In recent years, 4 RCTs are published that targeted r‐IFG (Breitling et al., 2016; Breitling et al., 2020; Breitling‐Ziegler et al., 2021; Westwood et al., 2021). While these studies benefited from robust experimental design (i.e., double‐blind RCT with follow‐up, behavioral, and physiological measures), they have several caveats that ambiguate the contribution of r‐IFG to ADHD pathophysiology. The protocol applied in the Breitling et al.’s (2016) study was possibly suboptimal in inducing the required electrical field in the target region according to modeling of the electrical current flow (Salehinejad et al., 2020). Their second study (Breitling et al., 2020) also suffered from different experimental procedures in the control and ADHD groups, and reduction of stimulation intensity to 50% in 3 out of 14 participants, and a low sample size. The only RCT with a relatively large sample size is recently published and found null effects of 15 r‐IFG tDCS + cognitive training on ADHD symptoms and neuropsychological performance (Westwood et al., 2021). One methodological issue with this work is the concurrent intervention with tDCS + cognitive training. Without having a “tDCS only” condition, it is not possible to disentangle efficacy of tDCS alone. Indeed, combining two interventions may even counterbalance efficacy of each other given that the acute and neuroplastic effects of tDCS vary during, right after, and longer after the stimulation (Agboada et al., 2019) and this might behave differently in the developing brain (Moliadze et al., 2015). Moreover, the cathode electrode in this study was placed on the left supraorbital, a region that is known for its contribution to hot executive functions and reward processing (Nejati et al., 2018; 2021). Future studies are needed to systematically investigate the role of r‐IFG in different stimulation protocols.

In addition to dlPFC and r‐IFG, the vmPFC seems another promising region, especially for hot executive dysfunctions with emotional/motivational valence (Salehinejad et al., 2021). Only one tDCS study investigated the contribution of this region and found it causally involved in hot executive dysfunctions of children with ADHD. Considering ADHD subtypes (Molavi et al., 2020) whose symptoms differ in the cognition–emotion spectrum, it might be interesting to study the role of this region with regard to subtype‐specific profiles in future tDCS studies of ADHD. Finally, it is noteworthy that ADHD had the lowest number of RCTs with multi‐session tDCS protocol (29.41%) in comparison to ASD (81.8%) and dyslexia (57.1%) and this gap needs to be addressed in the future for evaluating clinical efficacy of tDCS in ADHD.

In addition to the factors related to stimulation parameters and study design, external and interindividual factors are largely missed in tDCS studies in ADHD. The disorder subtype (i.e., inattentive, hyperactive, combined) is related to heterogeneous symptoms manifestation and is related to different functional structural brain abnormalities (2020; 2019 ), which means different stimulation protocols are needed for each subtype. So far, none of the studies have considered this. Furthermore, ADHD is related to sleep difficulties and the majority of children with ADHD have late chronotypes (i.e., eveningness) (Bijlenga et al., 2019; Coogan & Mcgowan, 2017). Recent works also show applying tDCS on circadian non‐preferred time (Salehinejad et al., 2021) and under sleep pressure (Salehinejad et al., 2022) can abolish the expected effect on cortical excitability, tDCS‐induced neuroplasticity, and cognitive functions. This should be considered especially for the therapeutic application of tDCS in ADHD that is associated with a more evening oriented circadian preference and sleep difficulties.

### 4.2 ASD

All RCTs in ASD reported an improving effect on at least one of the outcome variables. A major concern here, however, is the number of studies with robust experimental design (double‐blind RCT), which constitutes 36.3% of all studies (4 of 11). Nevertheless, all RCTs with both single‐ and double‐blinded designs reported promising results. An advantage of tDCS studies in ASD was the use of multi‐session design, which was the case in 81.8% of studies (9 of 11 studies). This is especially important for evaluating clinical efficacy of the intervention and might be one reason for positive changes across all tDCS studies in children with ASD. This is important as previous physiological studies have shown that tDCS neuroplastic effects can be boosted by repeated tDCS sessions over motor and prefrontal regions (Fregni et al., 2006; Ho et al., 2016; 2020). The left dlPFC stimulation is reported promisingly effective in reducing behavioral problems in ASD. The vmPFC and cerebellum were found effective in the reported studies and worth further investigation in future studies, especially for social cognition deficits in ASD.

Furthermore, in tDCS studies conducted on ASD, opposite stimulation polarity (anodal vs cathodal) is applied with beneficial effects. This should be considered with respect to target symptoms, stimulation parameters (intensity, duration, and repetition rate), and the excitatory/inhibitory dysbalance in ASD. Cathodal stimulation of the left dlPFC was theoretically assumed to mitigate hyperactive behavior and restore inhibition (Dâurso et al., 2015; GÃmez et al., 2017), while left dlPFC anodal stimulation was applied to compensate for left hemispheric hypoactivity. Nonetheless, the classical concept of anodal‐excitatory/cathodal‐inhibitory has been questioned by recent studies on the human motor cortex both in adults (Batsikadze et al., 2013; 2020) and children (Moliadze et al., 2018). The beneficial effect of cathodal tDCS over the left DLPFC reported in autism studies should thus be interpreted carefully with respect to mechanisms of action, as these stimulation protocols might indeed have an excitability‐enhancing effect.

### 4.3 Dyslexia

Although the number of tDCS studies in children and adolescents with dyslexia is lower compared to ADHD and ASD, their results are very promising and all of the studies have an RCT design. Moreover, 57.14% of the trials had a multi‐session design with improving effects on outcome variables indicating that tDCS can be of great clinical interest in children and adolescents with learning disorders. Bilateral temporoparietal regions including the TPJ and superior temporal gyrus are the most often targeted regions. One argument beyond targeting these regions with anodal left–cathodal right hemisphere is that the inhibition of the right temporoparietal cortex and the simultaneous facilitation of the left temporoparietal cortex might change an underlying imbalance that could be at the core of dyslexia (Turker & Hartwigsen, 2022). Studies that applied other non‐invasive brain stimulation techniques in dyslexia also found the left auditory cortex as a promising region for improving reading abilities (Marchesotti et al., 2020). Other promising cortical regions for targeting in tDCS studies are the left inferior frontal gyrus and anterior cingulate gyrus which show increased activation in those children with dyslexia with improvement in oral language ability (Temple et al., 2003) and are among the suggested tDCS protocols in dyslexia (Vicario & Nitsche, 2013). In sum, available evidence suggests promising effects of tDCS in developmental dyslexia. Nevertheless, randomized clinical trials with long‐term follow‐up measurements are required to establish the clinical efficacy of this intervention. Given the promising results, it would be tempting to investigate the efficacy of tDCS in other learning disorders (e.g., dyscalculia) as well as other cognitive deficits that characterize dyslexia.

### 4.4 Limitations of the studies and the filed

#### 4.4.1 Design‐related limitations

The major limitations of tDCS studies included in these 2021 three neurodevelopmental disorders can be categorized into design‐related limitations and protocol‐related limitations. The first design‐related limitation is the number of subjects, which is still limited in the majority of tDCS studies. Only 13 and 5 of the included studies (*N* = 35) have a sample size ≥20 and ≥30, respectively, in the group that received tDCS. This is especially important for evaluating clinical efficacy. Second, we need to have RCTs with double‐blind design and follow‐up measurements specifically for evaluating the clinical efficacy. This issue was more problematic in tDCS studies in ASD (27% of studies did not report blindness) and dyslexia (42% single‐blinded design) in which we also see more promising clinical effects (see Figure 3).

#### 4.4.2 Protocol‐related limitations

First, the most obvious limitation here is the use of suboptimal stimulation protocols. It is surprising that we still do not have any titration study that systematically investigates different parameters of stimulation (e.g., different intensities (e.g. 2021), duration, electrode configurations) in one homogeneous sample size. This is required for realistic evaluation of applied protocols, which was not the case so far in ADHD and ASD studies (Figure 3d). Applying adequate stimulation intensity and optimal electrode placement which delivers maximum electrical field to the target region is an issue that can be partially resolved by a‐priory modeling of current flow in the head. Only 5 of 17 tDCS studies in ADHD (Breitling et al., 2016; Breitling et al., 2020; Breitling‐Ziegler et al., 2021; Salehinejad et al., 2020; Soff et al., 2017) and 3 of 11 tDCS studies in ASD (Hadoush et al., 2020; Han et al., 2022; Salehinejad et al., 2021), and 1 of 7 tDCS studies in dyslexia (Rahimi et al., 2019) calculated and reported electric field modeling, which should be taken into account in future studies for designing a more optimal protocol. In this respect, it is important that the field establishes methodological guidelines and/or suggested stimulation protocols for examining clinical, cognitive, and physiological outcomes specifically for the pediatric population. Second, combining stimulation with other interventions with an assumption that concurrent interventions can have a synergistic improving effect is another issue that should be considered. In the end, considering large heterogeneity in these disorders due to different reasons, adopting an individualized, anatomically adapted stimulation protocol seems to be the promising way to go on in the field of tES application in neurodevelopmental disorders.

## 5 CONCLUSION

Taken together, current research provides preliminary evidence for the therapeutic potential of tDCS in ADHD, ASD, and dyslexia of childhood and adolescence. However, we still have a long way ahead to establish tDCS‐based interventions in the developing population. To this end, large‐scale RCTs and translational studies covering the range from basic neurophysiology to application in cognitive‐clinical neuroscience are required. Furthermore, stimulation protocols applied in the most‐studied neurodevelopmental disorders show that we need to develop symptom‐specific stimulation protocols that take disorder‐specific conditions into account. In this line, inter‐individual variabilities should be also considered, in line with a “personalized” approach in NIBS research. This is even more important in the developing brain, which undergoes broad and quick physiological changes. Adopting a personalized approach would allow us to purposefully target deficits and symptoms and apply tDCS in individuals that will likely respond to the treatment.

## CONFLICT OF INTEREST

The authors declare no conflict of interest.

## AUTHOR CONTRIBUTIONS

**Mohammad Ali Salehinejad**: conceptualization, methodology, writing – review & editing, writing – original draft; supervision; **Elham Ghanavati**: methodology, formal analysis, writing – review & editing; **Benedikt Glinski**: formal analysis, writing – review & editing; **Amir‐Homayun Hallajian**: formal analysis, writing – review & editing; **Anita Azarkolah**: conceptualization; writing – review & editing; supervision.

### PEER REVIEW

The peer review history for this article is available at https://publons.com/publon/10.1002/brb3.2724

## Data Availability

Data sharing is not applicable to this article as no new data were created or analyzed in this study. The data that support the findings are available in the manuscript.
